# Necdin Controls Proliferation of White Adipocyte Progenitor Cells

**DOI:** 10.1371/journal.pone.0030948

**Published:** 2012-01-23

**Authors:** Kazushiro Fujiwara, Koichi Hasegawa, Tsuyoshi Ohkumo, Hiroyuki Miyoshi, Yu-Hua Tseng, Kazuaki Yoshikawa

**Affiliations:** 1 Institute for Protein Research, Osaka University, Suita, Osaka, Japan; 2 BioResource Center, RIKEN Tsukuba Institute, Tsukuba, Ibaraki, Japan; 3 Research Division, Joslin Diabetes Center, Harvard Medical School, Boston, Massachusetts, United States of America; Brigham and Women's Hospital, United States of America

## Abstract

White adipose tissues are composed mainly of white fat cells (adipocytes), which play a key role in energy storage and metabolism. White adipocytes are terminally differentiated postmitotic cells and arise from their progenitor cells (preadipocytes) or mesenchymal stem cells residing in white adipose tissues. Thus, white adipocyte number is most likely controlled by the rate of preadipocyte proliferation, which may contribute to the etiology of obesity. However, little is known about the molecular mechanisms that regulate preadipocyte proliferation during adipose tissue development. Necdin, which is expressed predominantly in postmitotic neurons, is a pleiotropic protein that possesses anti-mitotic and pro-survival activities. Here we show that necdin functions as an intrinsic regulator of white preadipocyte proliferation in developing adipose tissues. Necdin is expressed in early preadipocytes or mesenchymal stem cells residing in the stromal compartment of white adipose tissues in juvenile mice. Lentivirus-mediated knockdown of endogenous necdin expression *in vivo* in adipose tissues markedly increases fat mass in juvenile mice fed a high-fat diet until adulthood. Furthermore, necdin-null mutant mice exhibit a greater expansion of adipose tissues due to adipocyte hyperplasia than wild-type mice when fed the high-fat diet during the juvenile and adult periods. Adipose stromal-vascular cells prepared from necdin-null mice differentiate *in vitro* into a significantly larger number of adipocytes in response to adipogenic inducers than those from wild-type mice. These results suggest that necdin prevents excessive preadipocyte proliferation induced by adipogenic stimulation to control white adipocyte number during adipose tissue development.

## Introduction

Obesity causes many serious diseases such as type 2 diabetes mellitus, cardiovascular diseases, hypertension, and certain types of cancer [1]. Fat mass expands by increasing the volume and/or the number of adipocytes in white adipose tissues (WATs). Although increased lipid storage in white adipocytes has been thought to be a major cause of fat mass expansion, recent studies have suggested that adipocyte number is also a key determinant for fat mass [2], [3]. The number of adipocytes in obese individuals is larger than that in lean individuals, a difference established during childhood and adolescence [2]. Furthermore, adipocyte number increases in certain depots even in human adults after excessive food intake [3]. Because adipocytes are differentiated from multipotent mesenchymal stem cells or preadipocytes residing in WATs [4], [5], [6], stimulated preadipocyte proliferation during WAT development may contribute primarily to an increase in adipocyte number. In mice, preadipocytes in the stromal-vascular (SV) compartment of WATs can proliferate in response to excessive calorie intake and differentiate into mature adipocytes [7]. Thus, understanding the mechanisms controlling preadipocyte proliferation may provide insights into the etiology and prevention of obesity and its associated pathologies.

Necdin was originally identified as a gene product induced in neurally differentiated embryonal carcinoma stem cells [8]. Necdin is strongly expressed in postmitotic cells such as neurons and skeletal myocytes [9], [10]. Expression of the necdin gene (*Ndn*) is controlled through genomic imprinting, a placental mammal-specific epigenetic mechanism [11], [12]. Necdin, like the retinoblastoma protein (pRb) family, strongly suppresses cell proliferation [13] and interacts with viral oncoproteins and cellular E2F family proteins [14], [15], [16]. Furthermore, necdin binds to the tumor suppressor protein p53 and inhibits p53-dependent apoptosis, whereas these two proteins suppress cell proliferation in an additive manner [17], [18]. These findings suggest that necdin serves as an endogenous anti-mitotic and anti-apoptotic protein in postmitotic cells [19]. It has recently been reported that necdin is also expressed in stem cells or undifferentiated progenitors residing in non-neural tissues such as mesoangioblast stem cells [20], brown adipocyte precursors [21], skeletal muscle satellite cells [22], hematopoietic stem cells [23], [24], and hepatic stellate cells [25]. Necdin has been suggested to be an endogenous mitotic suppressor in hematopoietic stem cells [23], [24]. These findings prompted us to investigate whether necdin functions as an intrinsic regulator of preadipocyte proliferation to control white adipocyte number during WAT development.

In this study, we show that necdin is expressed in mesenchymal stem cells or early preadipocytes residing in the SV compartment of WATs. Using two *in vivo* systems of *Ndn*-mutant and lentivirus-mediated *Ndn* knockdown mice, we demonstrate that reduction of endogenous necdin in preadipocytes results in a significant WAT expansion. Furthermore, we show that necdin-deficient SV cells treated with adipogenic inducers differentiate into increased populations of committed preadipocytes and mature adipocytes *in vitro*. Based on these findings, we propose that necdin acts as an intrinsic regulator of preadipocyte proliferation during WAT development.

## Results

### Necdin is expressed in WAT stromal cells *in vivo*

**Figure 1:**
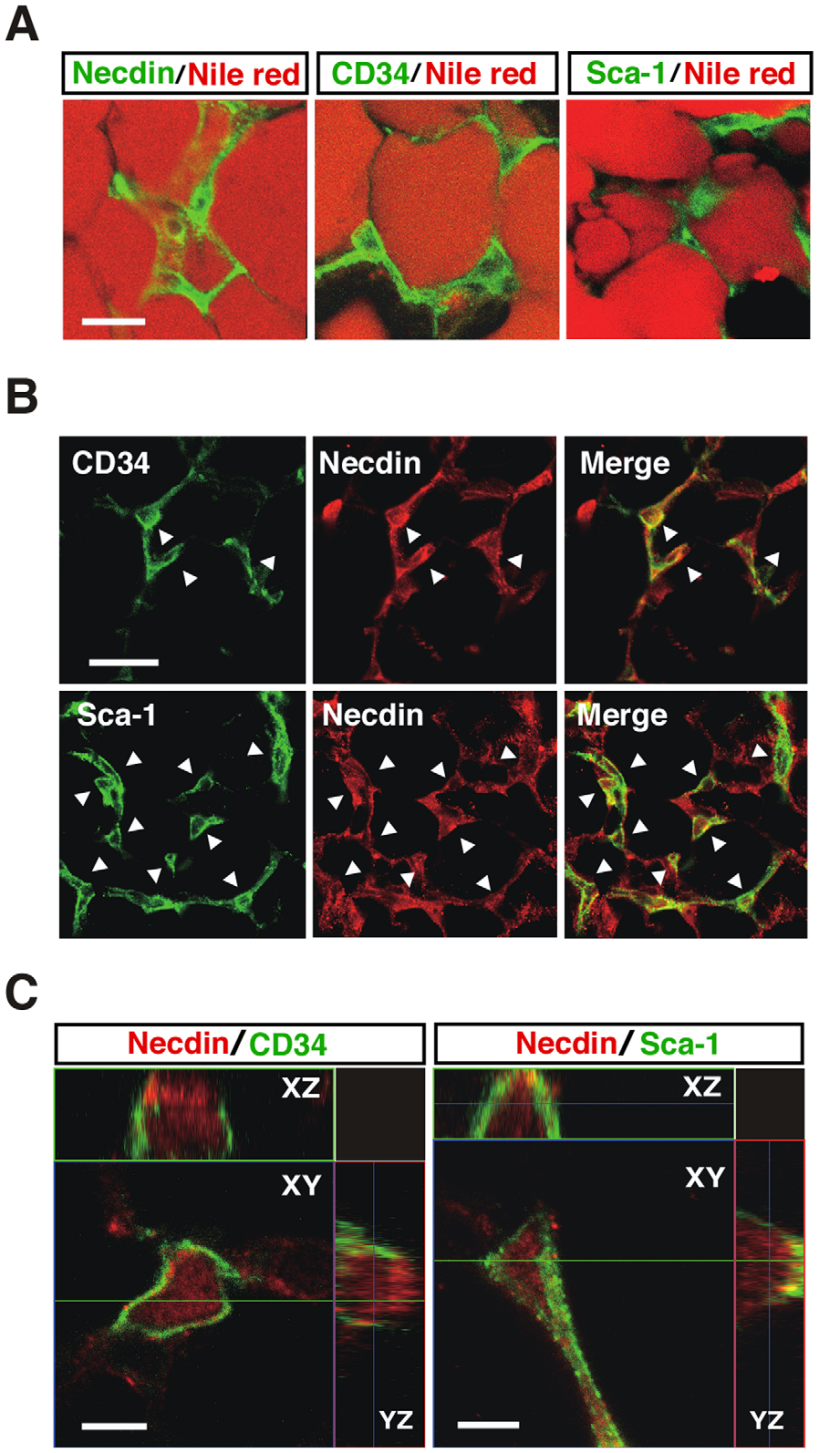
Necdin is expressed in adipose stromal cells *in vivo*. (*A*) Expression of necdin in WAT stroma. Interscapular WAT sections were prepared from 5-week-old male mice and co-stained with Nile red for mature adipocytes and antibodies against necdin, CD34 and Sca-1. (*B*) Expression of necdin in CD34^+^ and Sca-1^+^ cells. Arrowheads point to representative double-immunopositive cells. (*C*) Three-dimensional images of intracellular necdin, CD34 and Sca-1. Immunostained tissues were observed by multiple z-stack confocal laser-scanning microscopy. Accessory panels are along XZ and YZ axes. Scale bars; 20 µm (*A*), 10 µm (*B*), 5 µm (*C*).

The multipotent cells and preadipocytes are present in the stromal region of WATs and express stem cell markers such as CD34 and stem cell antigen-1 (Sca-1) [26]. Thus, we immunohistochemically examined the expression pattern of necdin and compared it with those of CD34 and Sca-1 in the WAT of 5-week-old mice (Fig. 1A). Necdin was expressed predominantly in WAT stromal cells, and its distribution pattern was similar to those of CD34^+^ and Sca-1^+^ cells. The number of necdin^+^ cells was larger than that of CD34^+^ or Sca-1^+^ cells in the WAT stroma, and virtually all of the stromal CD34^+^ and Sca-1^+^ cells overlapped with necdin^+^ cells (Fig. 1B). Three-dimensional confocal laser scanning microscopy for the subcellular localization of each protein revealed that necdin was localized mainly in the nucleus and moderately in the cytoplasm, whereas CD34 and Sca-1 were at or near the plasma membrane (Fig. 1C). These data suggest that necdin is expressed *in vivo* in WAT stromal cells that include early preadipocytes or mesenchymal stem cells.

### Lentivirus-mediated necdin downregulation enhances adipose expansion *in vivo*

**Figure 2:**
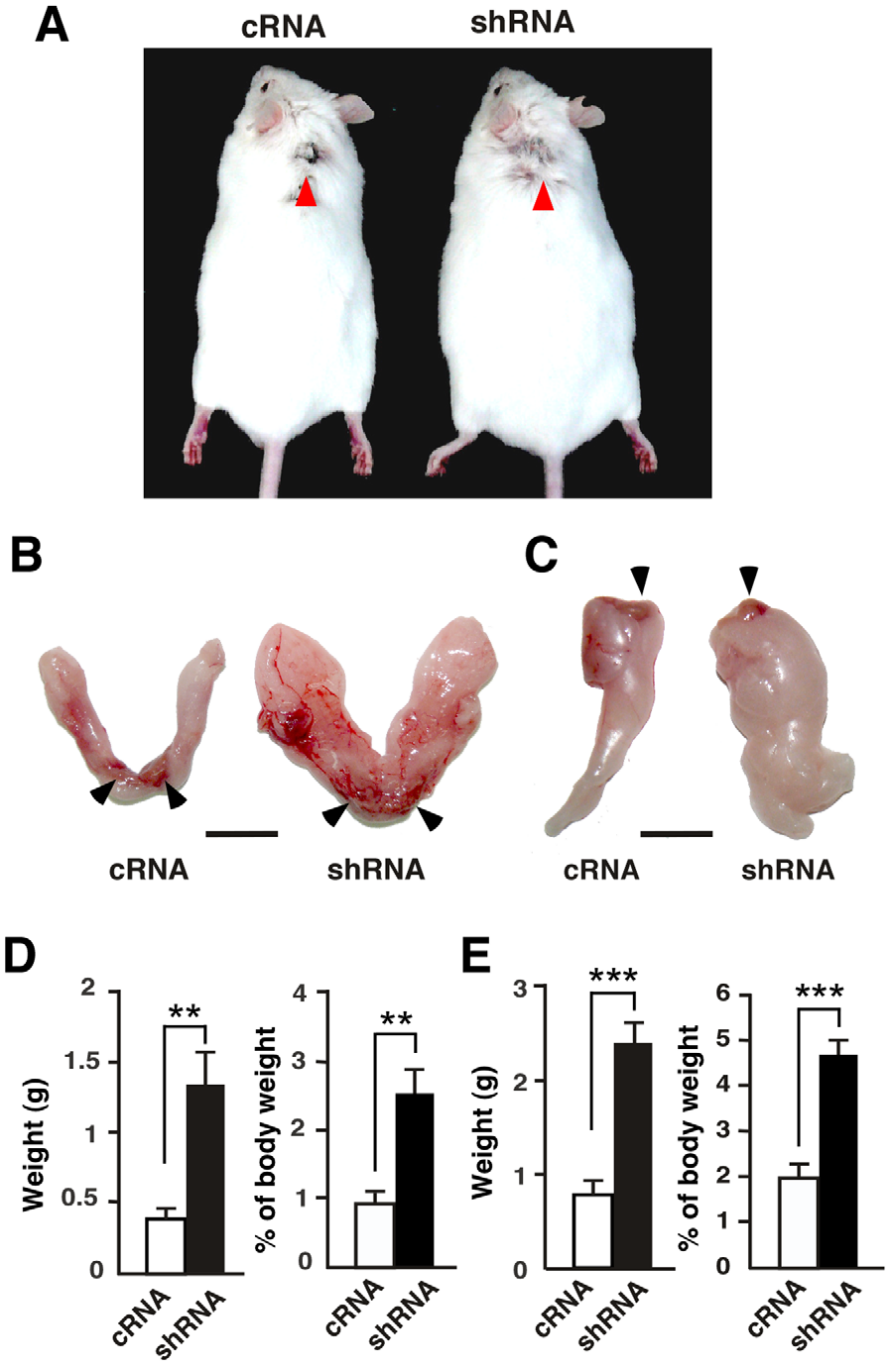
Lentivirus-mediated necdin knockdown enhances WAT expansion *in vivo*. (*A*) Representative mice infected with lentivirus vectors for control scrambled RNA (cRNA) and necdin shRNA (shRNA). Mice were infected with the recombinant lentiviruses at 5 weeks of age and subsequently fed a high-fat diet for 6 weeks. Red arrowheads point to the injection sites in the interscapular WAT. (*B*, *C*) Representative interscapular (*B*) and epididymal fat pads (*C*). WATs were excised from lentivirus-infected mice. Arrowheads point to brown fat depots (*B*) and testes (*C*) attached to the fat pads for orientation. Scale bars, 1 cm. (*D*, *E*) White fat pad weight. Interscapular (*D*) and epididymal (*E*) white fat pads excised from lentivirus-infected mice fed the high-fat diet were weighed after removing non-white-adipose tissues. Absolute (left) and relative values (right) are shown (mean ± SEM, *n* = 4). **P<0.01, ***P<0.001.

To examine the effect of necdin on preadipocyte proliferation, we attempted to downregulate its expression *in vivo* using a recombinant lentivirus that expresses necdin-specific short-hairpin RNA (necdin shRNA)(Fig. S1A). The necdin shRNA lentivirus was injected into the interscapular WAT near the brown adipose tissue (BAT) *in vivo* of 5-week-old mice, which were subsequently fed a high-fat diet for 6 weeks to induce preadipocyte proliferation (Fig. 2A). Surprisingly, the interscapular and epididymal fat pads (subcutaneous and visceral WATs, respectively) of mice injected with the necdin shRNA lentivirus had >3 times the weight of those of cRNA lentivirus-injected mice (Fig. 2B–E). We confirmed that necdin shRNA-expressing lentivirus downregulated expression of endogenous necdin in adipose SV cells both *in vitro* and *in vivo* (Fig. S1B, C). Necdin shRNA lentivirus-infected mice had a heavier body weight than cRNA lentivirus-infected mice (cRNA, 39±1.9 g; shRNA, 51±4.6 g; mean body weight ± SEM; *P*<0.05), suggesting that necdin knockdown *in vivo* enhances adiposity. These results suggest that endogenous necdin in the stromal preadipocytes *in vivo* prevents adipose expansion induced by high-fat diet intake.

**Figure 3:**
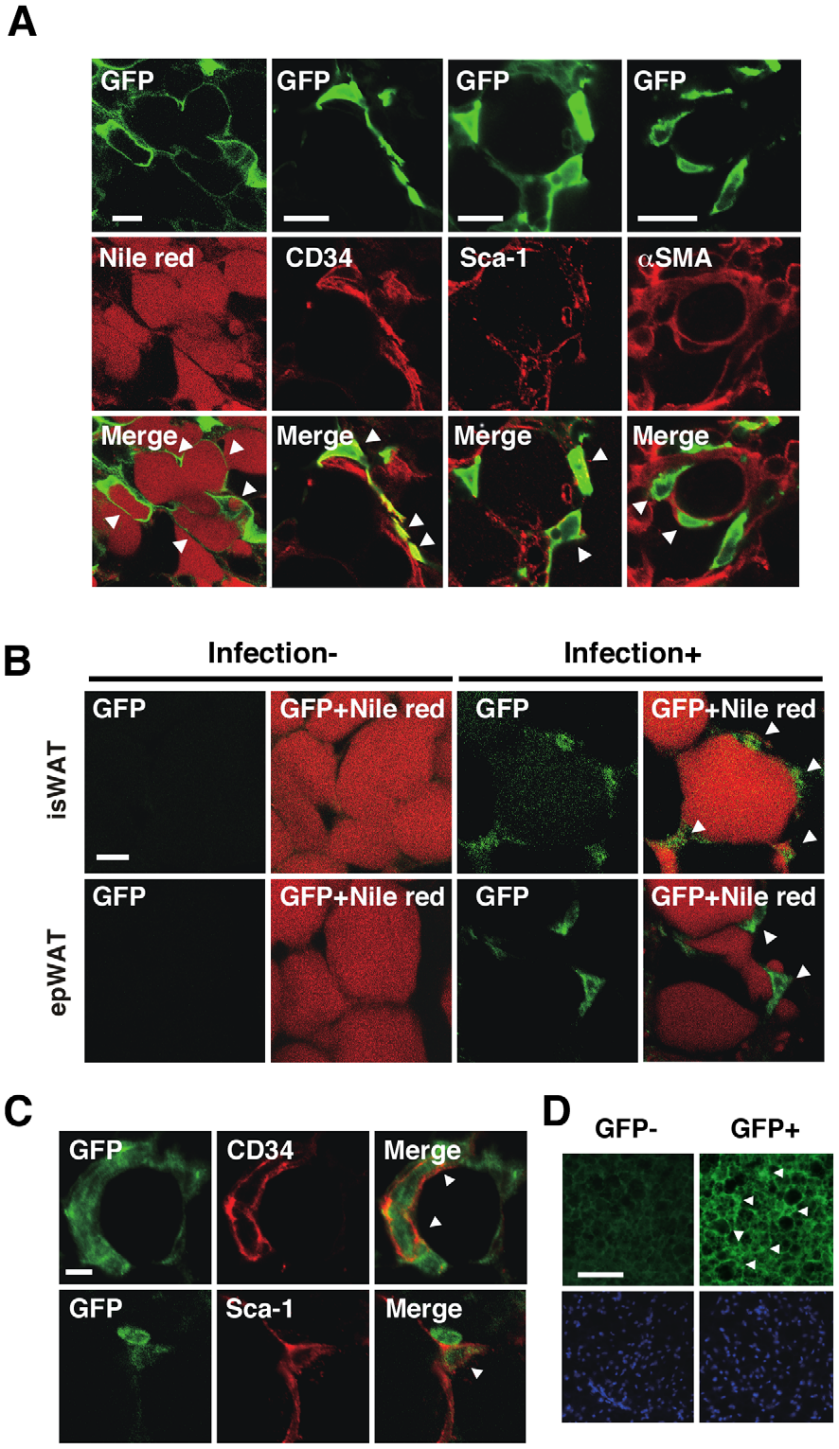
Lentivirus-infected WAT cells express GFP. (*A*) GFP expression in the interscapular WAT. GFP-expressing lentiviruses were injected into the interscapular fat pad of 5-week-old (P35) male mice. The interscapular WAT was fixed 48 hr after infection, co-stained for GFP and lipids (Nile red) or CD34, Sca-1, αSMA, and observed by confocal laser-scanning microscopy. Arrowheads (Merge) point to representative double-positive cells. (*B*) GFP expression in the WATs. The interscapular (isWAT) and epididymal WAT (epWAT) in mice infected with GFP-expressing lentivirus were co-stained with anti-GFP antibody and Nile red. Infection-, uninfected WATs (negative control); Infection+, infected WATs (6 weeks postinfection). Arrowheads point to GFP^+^ stroma cells. (*C*) GFP expression in CD34^+^ and Sca-1^+^ cells. Lentivirus-infected interscapular WATs were double-stained for GFP (green) and CD34 or Sca-1, and observed by confocal laser-scanning microscopy. Arrowheads (Merge) point to double-immunopositive stromal cells. (*D*) GFP expression in the BAT. Lentivirus-infected interscapular BAT was immunostained for GFP 6 weeks after infection and observed by fluorescence microscopy. Images stained with (GFP+) and without primary anti-GFP antibody (GFP-) (upper panels) and with Hoechst 33342 for nuclear DNA staining (lower panels) are shown. Scale bars; 20 µm (*A*), 10 µm (*B*, *C*), 100 µm (*D*).

To confirm that the recombinant lentiviruses infected the WAT stromal cells *in vivo*, we examined the expression pattern of green fluorescent protein (GFP), which is coexpressed with the lentivirus-mediated transgenes. GFP was expressed in Nile red-positive adipocytes and stromal cells expressing CD34^+^ or Sca-1^+^ 48 hr after viral infection (Fig. 3A). Vascular cells expressing α-smooth muscle actin (αSMA) in the WAT stroma also expressed GFP. In addition, some GFP^+^ cells were detected in the liver (data not shown), suggesting that the lentiviruses spread through blood from the injected site to other tissues. As expected, GFP was detected in the interscapular and epididymal WATs 6 weeks after viral infection (Fig. 3B). Most of the stromal cells expressing CD34 or Sca-1 overlapped with GFP^+^ cells in the WAT stroma (Fig. 3C), indicating that the lentiviruses efficiently infected stromal preadipocytes *in vivo*. Mature adipocytes expressed much lower GFP levels than those 48 hr after infection. GFP was also detected in brown adipocytes in the interscapular BAT 6 weeks after viral infection (Fig. 3D). These data indicate that lentivirus vectors efficiently deliver exogenous genes into the adipose tissue cells *in vivo*.

### Paternal Ndn-mutant mice exhibit enhanced adiposity

**Figure 4:**
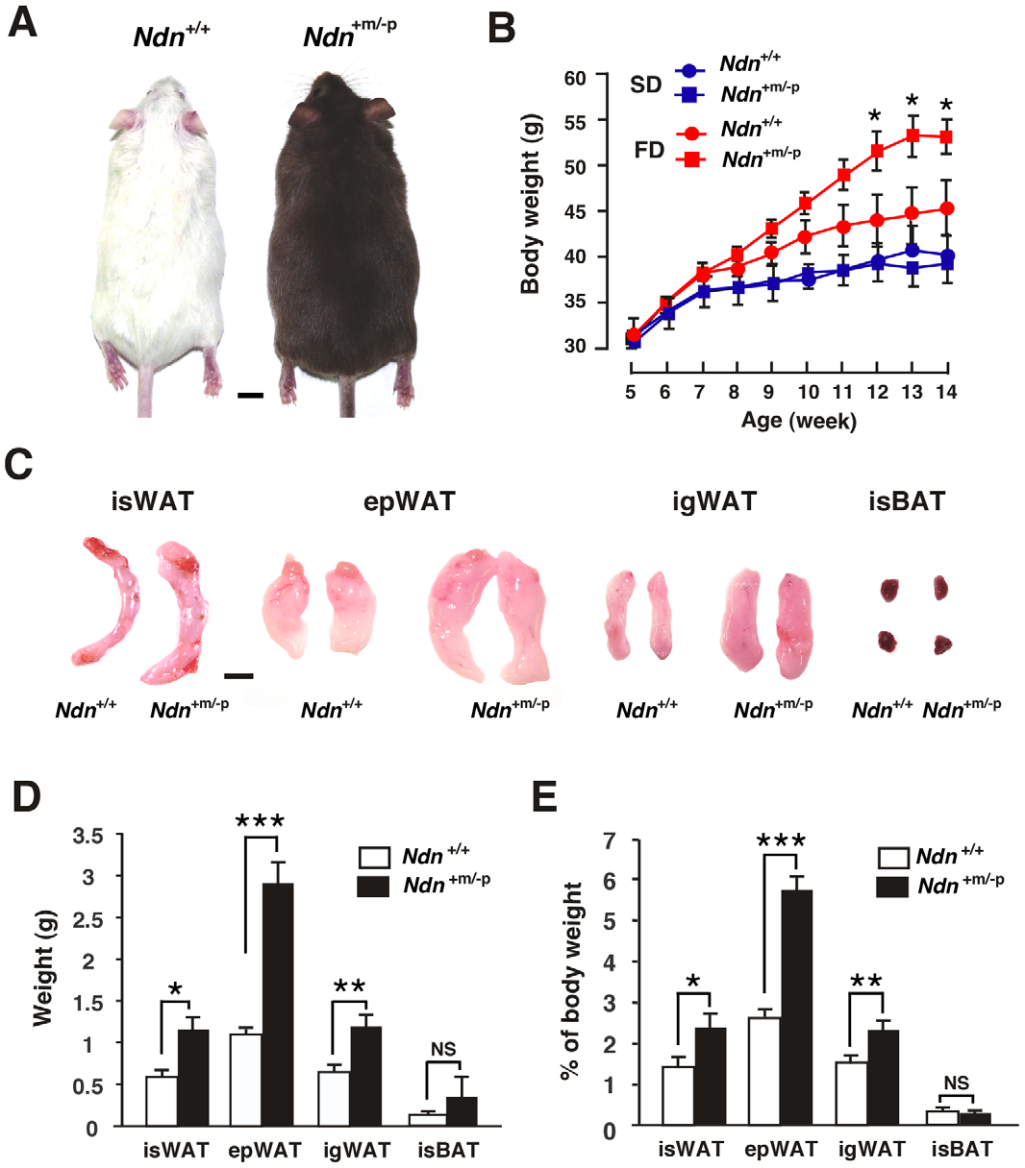
*Ndn* ^+m/−p^ mice exhibit enhanced adipose expansion. (*A*) Representative wild-type (*Ndn* ^+/+^, left) and paternal *Ndn*-mutated (*Ndn* ^+m/−p^, right) littermates. Mice were fed the high-fat diet from 5 to 14 weeks of age. Note that *Ndn* ^+m/−p^ mice carrying mutated *Ndn* linked to wild-type *pink-eyed dilution* gene exhibit a brown-hair phenotype on the ICR background. (*B*) Body weight curves of mice fed standard (SD) or high-fat diet (FD). Mouse body weight was measured weekly from 5 to 14 weeks of age (mean ± SEM, *n* = 5–8 for each group). *P<0.05, *Ndn* ^+m/−p^ vs. *Ndn* ^+/+^ using one-way ANOVA and Tukey's *post hoc* test. (*C*) Representative fat pads removed from high-fat-fed littermates. isWAT, interscapular WAT; epWAT, epididymal WAT; igWAT, inguinal WAT; isBAT, interscapular BAT. (*D, E*) Fat pad weight. Absolute (*D*) and relative values (*E*) are shown (mean ± SEM, *n* = 3–5). *P<0.05, **P<0.01, ***P<0.001. NS, not significant (*P*>0.05). Scale bars, 1 cm (*A, C*).

To investigate whether necdin deficiency affects WAT development *in vivo*, we used paternal *Ndn*-allele mutant (*Ndn* ^+m/−p^) male mice that were maintained on the ICR background [27]. These mice were fed the high-fat diet during the juvenile and adult periods (from 5 to 14 weeks of age) to stimulate preadipocyte proliferation. The mean body weight of *Ndn* ^+m/−p^ mice increased more than that of wild-type (*Ndn* ^+/+^) mice when fed the high-fat diet, whereas no significant difference in body weight was noted between *Ndn* ^+/+^ and *Ndn* ^+m/−p^ mice fed a standard diet (Fig. 4A, B). When pubertal male mice were fed the high-fat diet for 9 weeks (from 7 to 16 weeks of age), the difference in body weight gain between *Ndn* ^+/+^ and *Ndn* ^+m/−p^ mice was smaller than that of juvenile mice (Fig. S2A), indicating that weight gain rates of *Ndn* ^+m/−p^ mice are dependent on developmental stages. We found no significant difference in food intake between *Ndn* ^+/+^ and *Ndn* ^+m/−p^ mice during the 5-to-14 week period (Fig. S2B), eliminating the possibility that necdin deficiency enhances body weight gain due to hyperphagia. Furthermore, *Ndn* ^+m/−p^ mice exhibited neither abnormal body temperature (Fig. S2C) nor altered expression of energy metabolism-related genes in the skeletal muscle and liver, both of which expressed much lower levels of necdin than the brain at 5 weeks of age (Fig. S3A–C).

**Figure 5:**
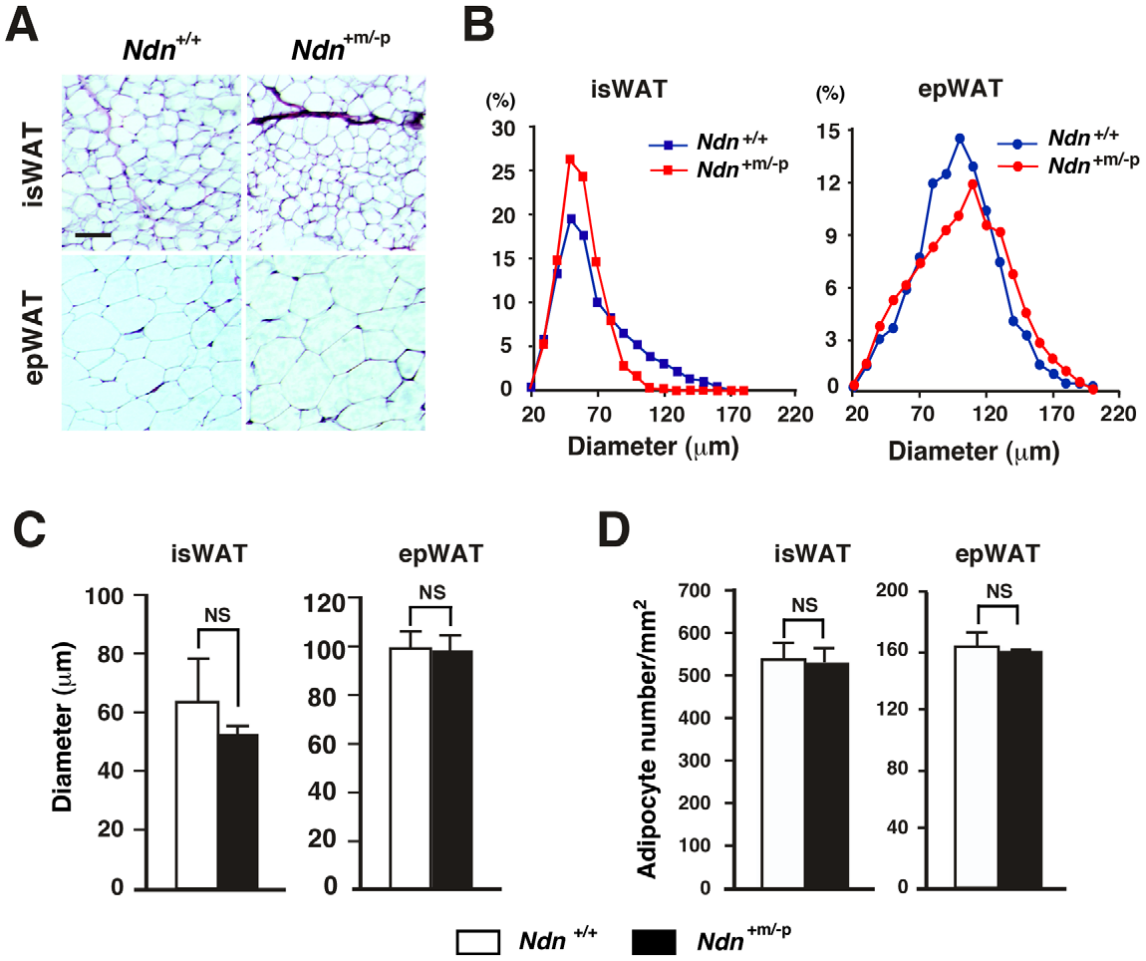
There is no difference in white adipocyte size between *Ndn* ^+/+^ and *Ndn* ^+m/−p^ mice. (*A*–*C*) Adipocyte size analyses. WATs of *Ndn* ^+/+^ and *Ndn* ^+m/−p^ littermates fed the high-fat diets from 5 to 14 weeks of age were embedded in paraffin. The WAT sections of 5 µm thickness were stained with hematoxylin and eosin (*A*). Adipocyte diameters in interscapular (isWAT) and epididymal WATs (epWAT) were measured using NIH Image J software, and shown as frequency distribution (*B*) and mean diameter ± SEM (*C*)(>400 cells per section, *n* = 3 for *Ndn* ^+/+^, *n* = 4 for *Ndn* ^+m/−p^). Scale bar, 100 µm. (*D*) Densities of adipocytes in WATs. Adipocyte densities in the stained sections (observed areas, 300–600 µm^2^) are presented as the number of adipocytes per square millimeter (examined 3 non-overlapping areas per slice, mean ± SEM, *n* = 3). NS, not significant (*P*>0.05) (*C*, *D*).

Remarkably, the interscapular, epididymal and inguinal WATs of *Ndn* ^+m/−p^ mice were significantly larger than those of *Ndn* ^+/+^ mice when fed the high-fat diet for 9 weeks (Figs. 4C–E and S4A, B). In contrast, there was little or no difference in BAT mass between *Ndn* ^+/+^ and *Ndn* ^+m/−p^ mice at 14 weeks of age. To examine whether necdin deficiency increases adipose mass by hypertrophy (increased volume) or hyperplasia (increased number) of white adipocytes, we measured the diameters of white adipocytes in the interscapular and epididymal WATs of *Ndn* ^+/+^ and *Ndn* ^+m/−p^ mice fed the high-fat diet (Fig. 5A, B). The diameters of epididymal WATs were larger than those of interscapular WATs in mice fed the high-fat diet, consistent with the previous findings that adipocytes of visceral WATs, but not of subcutaneous WATs, exhibit hypertrophy after high-fat diet intake [7]. However, mature adipocytes in the interscapular and epididymal WATs showed no significant difference in size and density between *Ndn* ^+/+^ and *Ndn* ^+m/−p^ mice (Fig. 5C, D), indicating that hyperplasia, rather than hypertrophy, contributes predominantly to the increased fat mass in *Ndn* ^+m/−p^ mice.

### Necdin deficiency enhances preadipocyte proliferation *in vitro*

**Figure 6:**
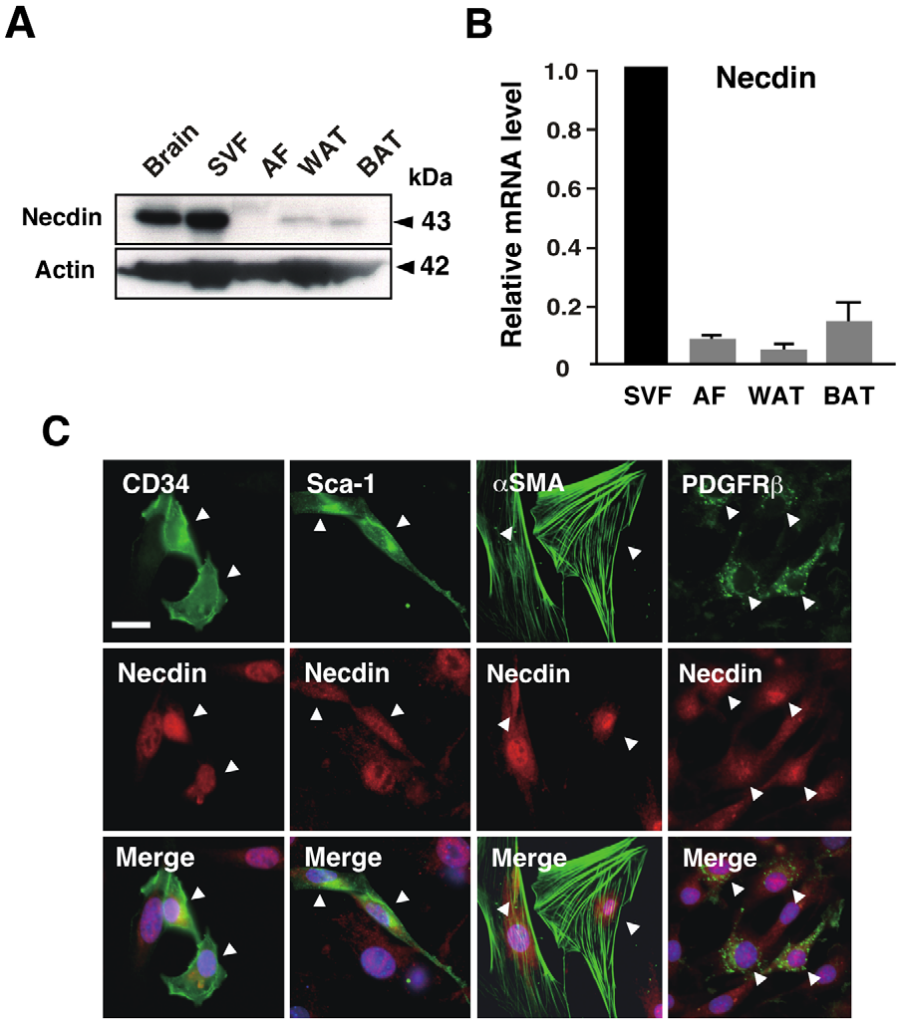
Necdin is expressed in primary adipose SV cells. (*A*) Western blot analysis of endogenous necdin. WAT stromal-vascular fraction (SVF), adipocyte fraction (AF), pooled WAT (WAT), interscapular BAT (BAT), and brain were prepared from 5-week-old mice. Necdin in the extracts was analyzed by Western blotting. Molecular sizes are in kilodaltons (kDa). (*B*) qRT-PCR for necdin mRNA. Total RNA was extracted from the above fractions and tissues. Necdin mRNA was analyzed by qRT-PCR. (*C*) Immunocytochemistry. Primary SV cells were prepared from 5-week-old mice, cultured, and stained by immunocytochemistry for necdin (red) and CD34, Sca-1, αSMA, or PDGFRβ (green). Double-stained images are merged with nuclear DNA staining (blue) (Merge). Arrowheads point to cells co-expressing necdin and the marker protein. Scale bar, 20 µm.

To examine whether necdin deficiency promotes preadipocyte proliferation in a cell-autonomous manner, we used primary SV cells prepared from WATs of *Ndn* ^+/+^ and *Ndn* ^+m/−p^ 5-week-old littermates. We first examined whether primary SV cells prepared from WATs express necdin. Western blot and quantitative reverse transcription-PCR (qRT-PCR) analyses revealed that primary SV cells expressed much higher necdin levels than the adipocyte-rich fraction (Fig. 6A, B). *CD34* mRNA was also highly expressed in the SV cells, in which expression of mRNAs for differentiated adipocyte markers such as peroxisome proliferator-activated receptor-γ2 (*PPARγ2*), adipocyte fatty acid binding protein (*aP2*), and uncoupling protein 1 (*UCP1*) was low (Fig. S5A–D). These data suggest that necdin is strongly expressed in the SV cells. Immunocytochemistry revealed that necdin was localized mainly in the nucleus of primary SV cells that express CD34 and Sca-1 (Fig. 6C). Necdin was also expressed in the subpopulations expressing the pericyte/vascular cell markers αSMA and platelet-derived growth factor receptor β (PDGFRβ) [28].

**Figure 7:**
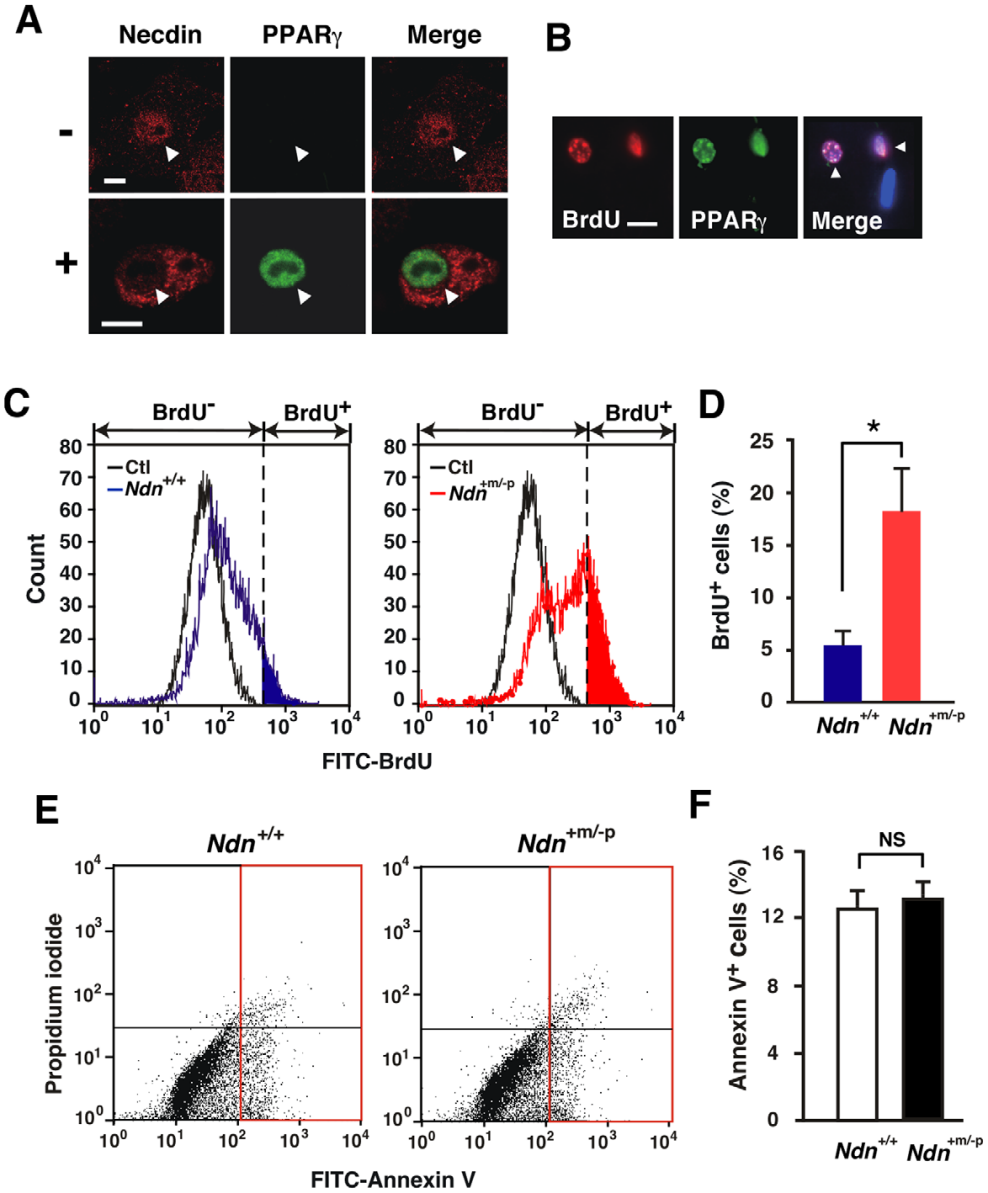
Necdin deficiency enhances preadipocyte proliferation in adipose SV cells. (*A*) Co-immunostaining for necdin and PPARγ. SV cells were treated with (+) or without (−) adipogenic inducers, and fixed 72 hr after induction. Cells were double-immunostained for necdin (red) and PPARγ (green), and confocal laser microscopic images are merged (Merge). Arrowheads point to the nucleus. (*B*) Co-staining for BrdU and PPARγ. SV cells were treated with adipogenic inducers, pulse-labeled with BrdU, triple-stained for BrdU, PPARγ and nuclear DNA with Hoechst 33342, and observed by fluorescence microscopy. Arrowheads (Merge) point to BrdU^+^ PPARγ^+^ cells. (*C, D*) Flow cytometry for BrdU incorporation. SV cells were prepared from *Ndn* ^+/+^ and *Ndn* ^+m/−p^ mice, treated with adipogenic inducers, and analyzed by flow cytometry for BrdU incorporation. BrdU^+^ cells in the colored area (blue for *Ndn* ^+/+^, red for *Ndn* ^+m/−p^)(*C*) were counted (*D*). The threshold (broken line) was set using negative control cells without BrdU treatment (Ctl). (*E, F*) Flow cytometry for apoptosis. SV cells were treated with adipogenic inducers and analyzed 20 hr later by flow cytometry using FITC-labeled Annexin V. *P<0.05. NS, not significant (*P*>0.05). Scale bars; 10 µm (*A*), 20 µm (*B*).

We then analyzed the subcellular localization of necdin in the SV cells during adipogenic differentiation by immunocytochemistry using PPARγ as a differentiation marker. Necdin was localized in the nucleus of undifferentiated PPARγ^−^ cells and the cytoplasm of differentiated PPARγ^+^ cells, suggesting that the nucleocytoplasmic translocation of necdin occurs during adipogenic differentiation (Fig. 7A). Because the anti-PPARγ antibody used in this experiment also recognizes PPARα and PPARβ/δ, we analyzed mRNA expression of three PPAR subclasses by quantitative RT-PCR and found that PPARα and PPARβ/δ mRNA levels in the SV cell-derived adipocytes were 15% and 21%, respectively, of the PPARγ (PPARγ1/2) level, indicating that PPARγ is expressed predominantly during adipogenesis of the SV cells.

We next determined the cell number during adipogenic differentiation of the SV cells (Fig. S6). The number of necdin-deficient SV cells was larger at each time point than that of wild-type control cells, suggesting that endogenous necdin in the SV cells suppresses their proliferation. Because only the adipogenic subpopulation of SV cells is expected to differentiate into adipocytes, we measured the number of newly generated adipocytes by bromodeoxyuridine (BrdU) incorporation assay combined with PPARγ immunocytochemistry, an *in vitro* system used for adipogenic mitotic clonal expansion [29]. Virtually all of the BrdU^+^ cells overlapped with PPARγ-expressing cells (Fig. 7B), indicating that adipocytes are newly generated through preadipocyte proliferation upon adipogenic induction. Flow cytometric analysis for BrdU incorporation revealed that necdin-deficient SV cells contained a significantly larger number of BrdU^+^ cells than control SV cells after adipogenic induction (Fig. 7C, D). On the other hand, no significant increase in apoptosis was observed in necdin-deficient SV cells treated with adipogenic inducers as analyzed by flow cytometry for Annexin V binding (Fig. 7E, F). These data suggest that preadipocyte proliferation is enhanced in necdin-deficient SV cells during adipogenesis without affecting their viability.

### Necdin deficiency enhances adipocyte differentiation *in vitro*

**Figure 8:**
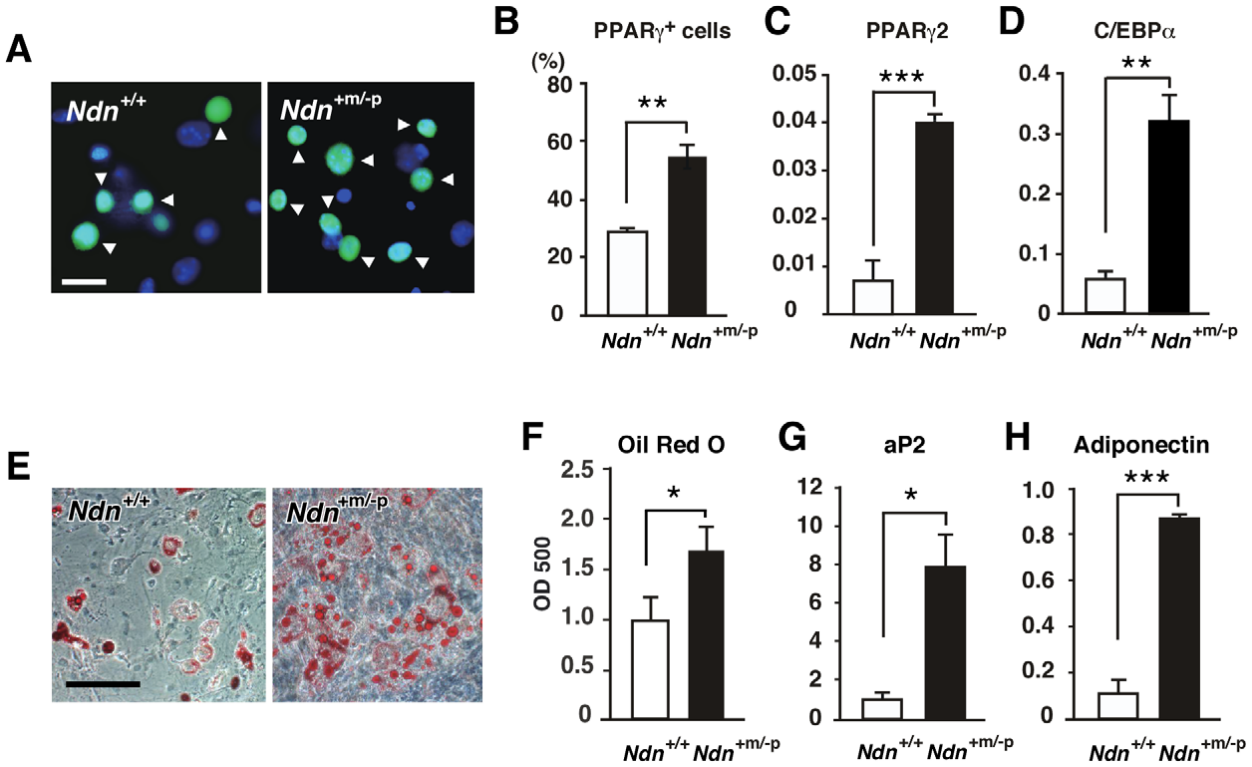
Necdin deficiency enhances adipocyte differentiation in adipose SV cells. (*A*, *B*) PPARγ^+^ cells. SV cells prepared from *Ndn* ^+/+^ and *Ndn* ^+m/−p^ littermates were treated with adipogenic inducers and double-stained for PPARγ (green) and nuclear DNA (blue)(*A*). PPARγ^+^ cells (arrowheads in *A*) were counted (mean ± SEM, *n* = 3)(*B*). (*C, D*) Expression levels of *PPARγ2* and *C/EBPα* mRNAs. *PPARγ2* (*C*) and *C/EBPα* (*D*) mRNA levels were analyzed by qRT-PCR 72 hr after adipogenic induction. (*E, F*) Oil Red O-staining. SV cells were stained with Oil Red O 8 days after adipogenic induction (*E*), and intracellular Oil Red O was quantified by spectrophotometry (mean ± SEM, *n* = 4–6)(*F*). (*G, H*) Expression levels of *aP2* and *adiponectin* mRNAs. The *aP2* (*G*) and *adiponectin* (*H*) mRNA levels were analyzed by qRT-PCR 8 days after adipogenic induction (mean ± SEM, *n* = 3). All mRNA levels (*C, D, G, H*) are shown as relative values to *GAPDH* mRNA levels (mean ± SEM, *n* = 3) *P<0.05, **P<0.01, ***P<0.001. Scale bars; 20 µm (*A*), 100 µm (*E*).

We then examined whether adipocyte differentiation is facilitated in necdin-deficient SV cells after adipogenic induction. Necdin-deficient SV cells contained a significantly higher PPARγ^+^ population than wild-type cells after adipogenic induction (Fig. 8A, B). Concurrently, *PPARγ*2 and *CCAAT/enhancer-binding protein α* (*C/EBPα)* mRNA levels were markedly increased (Fig. 8C, D). We also examined whether these SV cells are capable of differentiating into mature adipocytes. Necdin-deficient SV cells prepared from *Ndn* ^+m/−p^ mice differentiated into more Oil Red O^+^ adipocytes than control SV cells 8 days after adipogenic induction (Fig. 8E, F). In these differentiated adipocytes *in vitro*, mRNA levels of the mature adipocyte markers *aP2* and *adiponectin* were also markedly increased (Fig. 8G, H). These data suggest that necdin deficiency increases the number of mature adipocytes owing to the enhanced preadipocyte proliferation in response to adipogenic induction.

## Discussion

The present study has shown that necdin functions as an intrinsic suppressor of preadipocyte proliferation both *in vivo* and *in vitro* to control the adipocyte number during WAT development. We found that lentivirus-mediated delivery of necdin shRNA induced a marked expansion of subcutaneous and visceral WATs *in vivo*, suggesting that early preadipocytes or mesenchymal stem cells in the stromal compartment of WATs are highly susceptible to lentivirus infection. Thus, the lentivirus-mediated gene transfer may be a promising tool for modulating endogenous gene expression *in vivo* in early adipocyte progenitors of WATs. We also found that *Ndn* ^+m/−p^ mice fed the high-fat diet during the juvenile and adult periods exhibited a significant expansion of subcutaneous and visceral fat depots due to adipocyte hyperplasia. We assume that preadipocytes can proliferate and differentiate into mature adipocytes in response to adipogenic stimuli such as high-calorie diets during the juvenile period. Our present data implicate that necdin suppresses the high-calorie-stimulated proliferation of preadipocytes *in vivo* during the critical period to control white adipocyte number in WATs.

We found that necdin was expressed in WAT SV cells expressing CD34^+^ and Sca-1^+^, both of which are markers of mesenchymal stem cells or preadipocytes [26]. Furthermore, SV cells other than these CD34^+^ Sca-1^+^ cells expressed necdin *in vivo* and *in vitro* (Figs. 1B and 6C). Necdin was also expressed in αSMA^+^ and PDGFRβ^+^ vascular cells (Fig. 6C), which potentially differentiate into adipocytes [28]. However, we found it difficult to identify specific necdin-expressing SV cell types in a more accurate manner. Although many cell markers have been used for identification of specific cell types during adipogenesis, most of them are also expressed in other cell lineages [5]. In addition, expression levels of these markers vary at different stages of adipogenic differentiation [5]. We found that necdin-expressing SV cells differentiate not only into adipocytes but also into osteocytes, chondrocytes, myocytes, and neuron-like cells (unpublished observations). Thus, we assume that necdin is expressed in a variety of undifferentiated multipotent cells residing in the WAT stroma and serves as an intrinsic suppressor of their proliferation and differentiation.

In the present study, a significant acceleration of preadipocyte proliferation was observed in necdin-deficient SV cultures during adipogenic differentiation. This assay system has been often used for the evaluation of adipogenic differentiation in adipose SV cells [7], [26]. Because PPARγ, a major regulator of adipogenesis, is a representative marker for adipogenic differentiation, we used PPARγ for immunocytochemical analyses of differentiating SV cells. Although the antibody used in the present study recognizes other PPAR subclasses, we confirmed by qRT-PCR that PPARγ is the major PPAR species expressed in SV cell-derived adipocytes. Using immunocytochemistry for necdin and PPARγ, we were able to demonstrate the nucleocytoplasmic translocation of necdin during adipogenic differentiation of the SV cells (Fig. 7A). We speculate that nuclear necdin suppresses proliferation of undifferentiated preadipocytes and its translocation into the cytoplasm triggers the proliferation and differentiation of preadipocytes. We are now investigating the molecular mechanism underlying the translocation of necdin through its posttranslational modifications. In necdin-deficient SV cultures treated with adipogenic inducers, the enhancement of preadipocyte proliferation resulted in the increased population of mature adipocytes. In contrast, necdin is indispensable for the viability of nascent neurons which abundantly express necdin and undergo apoptosis in the absence of necdin [16], [18], [27]. These findings implicate that necdin suppresses preadipocyte proliferation and subsequent adipocyte differentiation but is dispensable for the viability of differentiated adipocytes, which express low levels of endogenous necdin (Fig. 6A, B).

We found that BAT mass was unchanged in the paternal *Ndn* mutant mice in the present experimental context, although necdin suppresses brown adipocyte differentiation *in vitro* [21]. This may be because white and brown adipocytes differentiate through distinct pathways in which different regulatory factors are involved [5], [30], [31], [32]. BAT expansion is promoted by cold stimulation and catecholamine treatment [33]. Furthermore, preadipocytes switch their fate from white to brown adipocytes in the absence of pRb family members [34], [35], [36], suggesting that pRb family proteins contribute primarily to the fate choice of white adipocyte lineage commitment rather than the control of white adipocyte number *in vivo*. Thus, necdin, unlike the pRb family members, may specifically suppress white preadipocyte proliferation induced by adipogenic stimulation without affecting the cell fate.

Necdin expression is absent in patients with Prader-Willi syndrome (PWS), a classic genomic imprinting-associated disorder that is frequently accompanied by hyperphagia and early-onset morbid obesity [11], [12]. PWS is caused by the lack of paternal expression of several contiguous genes including *NDN* located in chromosome 15q11-q13 region, which has syntenic homology with mouse chromosome 7C region. Mice bearing mutations in this region [37], [38], [39] exhibit neonatal lethality on a C57BL/6 background, indicating that neonatal lethality is a common phenotype in these PWS model mice. Necdin-deficient mice also exhibit neonatal lethality on the C57BL/6 background [40], [41], whereas necdin-deficient mice on the ICR background exhibit no lethality throughout the lifetime [27]. In contrast, C57BL/6 mice deficient in the C/D box small nucleolar RNA *Snord116* (*Pwcr1*/*MBII-85*), one of the PWS-associated contiguous genes, develop hyperphagia in adulthood but stay lean even on a high-fat diet [42]. Thus, it might be interesting to examine the phenotypes of mutant mice deficient in both *necdin* and *Snord116*.

The present study provides novel insights into the pathogenesis of PWS-associated obesity as it has been widely accepted that morbid obesity in PWS patients is attributable solely to hyperphagia due to hypothalamic defects. In view of the present experimental results, we speculate that PWS patients gain larger numbers of white adipocytes in WATs than normal subjects during the critical period for adipocyte number determination. It has recently been reported that PWS patients exhibit higher insulin sensitivity than body mass index-matched obese controls [43]. Furthermore, there is evidence suggesting that a limited WAT expansion predisposes to insulin resistance in patients with type 2 diabetes mellitus [44], [45]. These findings implicate that the increased number of adipocytes elevates the insulin sensitivity in PWS patients. Further studies on adipocyte number, size, and density in the WATs of PWS patients are required to substantiate the adipocyte hyperplasia in PWS patients.

Various signal transduction events in the brain and peripheral organs can change the overall energy balance that causes or prevents obesity [46]. Thus, excessive food intake and reduced energy expenditure in PWS patients may exacerbate the accumulation of white adipocytes in WATs under necdin-deficient conditions. This indicates that the preadipocyte abnormality in combination with the hypothalamic defect may contribute to the morbid obesity in PWS patients. Necdin is strongly expressed in the brain but very weakly in peripheral non-neuronal tissues such as muscle and liver (Fig. S3). We found transient upregulation of neuropeptide gene expression in the hypothalamic arcuate nucleus during the early post-weaning period, but no significant differences in *ad libitum* food intake and net energy expenditure were observed between necdin-null and wild-type mice during the juvenile and adult periods (unpublished observations). Thus, it seems unlikely that the hypothalamic control of energy metabolism is involved in the adipocyte hyperplasia in necdin-null mice. Besides pathological conditions such as PWS, necdin is likely to control preadipocyte proliferation to set the adipocyte number during normal WAT development. Further studies on the mechanisms underlying necdin-regulated preadipocyte proliferation will expand our knowledge on adipocyte number determination and its abnormality leading to obesity and associated pathologies.

## Materials and Methods

### Ethics statement

This study was approved by the Animal Experiment Committee (Approval No. 19-07-0) and Recombination DNA Committee (Approval No. 2938-1) of Institute for Protein Research, Osaka University, and performed in accordance with institutional guidelines.

### Immunohistochemistry

WATs were removed from 5-week-old (postnatal day 36)(P36) male ICR mice, fixed in 4% formaldehyde, incubated at 4°C with 15% and 30% sucrose for 12 hr and 24 hr, respectively, and embedded in OTC compound (Sakura Finetechnical). Frozen tissues were sectioned at 10–16 µm thickness with a cryostat (CM1850; Leica) and air-dried for 1 hr at room temperature (RT). Dried sections were washed with PBS-Tween 20 (0.05%) and incubated for 5 min at RT. Sections were incubated with PBS containing 1% BSA for 30 min at RT and incubated at 4°C overnight with the primary antibodies against necdin (NC243) [47], CD34 (RAM34; eBioscience), Sca-1 (D7; eBioscience), and GFP (MBL). Sections were incubated for 1 hr at RT with secondary antibodies against IgGs conjugated with cyanine 2, cyanine 3 (Jackson ImmunoResearch) and Alexa 488 (Molecular Probes), and mounted with SlowFade Antifade reagent (Molecular Probes). Intracellular lipids in WAT sections were stained with Nile red (100 ng/ml, Molecular Probes). Images were observed with a confocal laser-scanning microscope (LSM5 Pascal, Carl Zeiss MicroImaging). Three-dimensional images were obtained by multiple z-stack confocal laser-scanning microscopy.

### Lentivirus vectors

The miRNA expression vector was constructed using BLOCK-iT Pol II miR RNAi Expression Vector Kit (Invitrogen) and synthetic oligonucleotides (necdin miR: forward: 5′-TGCTGTAATTCTGCTGGACGAACTCCGTTTTGGCCACTGACTGACGGAGTTCGCAGCAGAATTA-3′, reverse: 5′-CCTGTAATTCTGCTGCGAACTCCGTCAGTCAGTGGCCAAAACGGAGTTCGTCCAGCAGAATTAC -3′). Control miRNA expression vectors were constructed using pcDNA6.2-GW/EmGFP-miR-neg control plasmid (Invitrogen) and oligonucleotide: 5′-GAAATGTACTGCGCGTGGAGACGTTTTGGCCACTGACTGACGTCTCCACGCAGTACATTTT -3′. These miRNA regions were transferred to the lentivirus vector CSII-EF-RfA to construct self-inactivation (SIN) vector plasmid (CSII-EF-miR-necdin, CSII-EF-miR-neg). The VSV-G-pseudotyped HIV vectors were generated by transient cotransfection of SIN plasmids with the packaging construct pCAG-HIVgp and the VGV-G-expressing construct pCMW-VSV-G-RSV-Rev into 293T cells [48]. High-titer stocks of HIV vectors were prepared by ultracentrifugation, and the titer was determined by the frequency of EmGFP^+^ 293FT cells. For viral infection *in vivo*, lentiviruses (2.4×10^7^ infection unit in 15 µl) were administered through a direct-vision injection into the interscapular fat pad near the BAT in 5-week-old (P36 or P37) male mice, which were subsequently fed the high-fat diet for 6 weeks. Infected tissues were analyzed by immunohistochemistry 48 hr and 6 weeks after lentivirus infection (1.2×10^7^ and 2.4×10^7^ infection unit, respectively). Cells were double-stained with Nile red and antibodies against GFP, CD34, Sca-1 and αSMA (1A4, Sigma-Aldrich).

### *Ndn* mutant mice

Mice bearing a targeted mutant *Ndn*-allele (*Ndn^tm1Ky^*) were generated, bred, and genotyped as described [27]. Male *Ndn* ^+m/−p^ were maintained on the ICR background and crossed with wild-type ICR females to obtain wild-type (*Ndn* ^+/+^) and necdin-deficient (*Ndn* ^+m/−p^) littermates. *Ndn* ^+/+^ and *Ndn* ^+m/−p^ 5-week-old male (P36 or P37) littermates were fed a standard diet (D12405B containing 70% carbohydrate, 20% protein and 10% fat, Research Diet) or a high-fat diet (D12492 containing 20% carbohydrate, 20% protein and 60% fat, Research Diets) for 9 weeks. The body weight and food intake were measured weekly.

### Primary SV cells

Adipose SV cells were isolated from pooled interscapular, inguinal, and epididymal WATs of 5-week-old (P36 or P37) male mice as previously reported [28]. WATs were digested with 1 mg/ml collagenase Type IV (Sigma-Aldrich), filtered through sterilized lens paper, and centrifuged. Enriched adipocytes floating on the top were removed or collected as the adipocyte fraction. Cell pellets were used as SV cells and cultured in DMEM supplemented with bFGF (10 ng/ml, PeproTech) and 10% FBS.

### Western blotting

Cell fractions and tissues were prepared from 5-week-old male mice. The lysates (10 µg protein) were separated by 7.5% SDS-PAGE, transferred onto Immobilon membrane (Millipore), and blotted with antibodies against necdin (NC243), actin (JLA20, DSHB), and β-tubulin (Sigma-Aldrich). Membranes were incubated with peroxidase-conjugated anti-rabbit and anti-mouse IgGs (Cappel), and proteins were visualized with chemiluminescence reagents (Perkin-Elmer). Molecular sizes are in kilodaltons (kDa).

### qRT-PCR

Total RNA was extracted with Trizol reagent (Invitrogen), and genomic DNA was digested with DNase I (Promega). cDNA was synthesized using Transcriptor First Strand cDNA synthesis kit (Roche). RT-PCR products were quantified using a real-time PCR instrument (LightCycler, Roche) and FastStart DNA MasterPLUS SYBR green I kit (Roche). Initial preheating step for 10 min at 95°C was followed by 45 cycles of touchdown PCR (10 sec at 95°C, 10 sec at 68°C to 58°C by 0.5°C per cycle and 10 sec at 72°C) and melting-curve analysis (at 50°C to 98°C). PCR primers used for mouse mRNA sequences are in Table S1. Relative values of mRNAs were obtained by normalizing with those of GAPDH mRNA unless stated otherwise.

### Immunocytochemistry

Cultured adipose SV cells were fixed in 4% formaldehyde for 10 min at RT and washed 3 times with PBS. Fixed cells were incubated with PBS containing 1% digitonin for 5 min on ice, washed 3 times with PBS, and incubated with PBS containing 1% BSA for blocking for 30 min at RT and incubated overnight at 4°C with primary antibodies against necdin (NC243), CD34 (RAM34), Sca-1 (D7), αSMA (1A4), PDGFRβ (APB5, eBioscience), and PPARγ (sc-7273, Santa Cruz Biotechnology). For double-immunostaining for BrdU and PPARγ, cells were labeled for 2 hr with 30 µg/ml BrdU (Sigma-Aldrich) and fixed 48 hr later with 10% formalin. Fixed cells were treated with 2 N HCl for 15 min at 37°C, neutralized with 0.1 M borate buffer (pH 8.5) for 20 min at RT and blocked with PBS containing 1% BSA for 30 min at RT. Cells were incubated overnight with primary antibodies against BrdU (BU1/75, Abcam) and PPARγ in PBS containing 1% BSA at 4°C overnight and with secondary antibodies against IgGs conjugated with cyanine 2, cyanine 3 (Jackson ImmunoResearch) and Alexa 488 (Molecular Probes). Cells were counterstained with Hoechst 33342 (Sigma-Aldrich) and mounted with the SlowFade Antifade reagent. Images were observed by fluorescence microscopy with Axioplan 2 and AxioCam camera system (Carl Zeiss MicroImaging).

### Adipogenic differentiation

SV cells (5×10^5^ cells/35 mm dish) at passage 3 were induced to differentiate using adipogenic chemicals [26]. Briefly, SV cells were grown to reach confluence and treated with 1 µM rosiglitazone (Alexis Biochemicals), 10 µg/ml insulin (Sigma-Aldrich), 1 µM dexamethasone (Sigma-Aldrich) and 500 µM isobutylmethylxanthine (Sigma-Aldrich). After adipogenic induction for 48 hr, cultures were maintained in DMEM containing 10% FBS, 1 µM rosiglitazone and 10 µg/ml insulin. Differentiated adipocytes were stained with 0.2% Oil Red O (Sigma-Aldrich) in 60% isopropyl alcohol and observed with an inverted microscope (IX70, Olympus). The amount of intracellular lipid-bound Oil Red O was measured by spectrophotometry at 500 nm by dissolving stained oil droplets in 100% isopropyl alcohol.

### Flow cytometry

For BrdU incorporation assay, SV cells treated with adipogenic inducers for 18 hr were labeled with 30 µg/ml BrdU for 2 hr and immunostained with FITC-labeled anti-BrdU antibody using BrdU Flow kit (BD Biosciences). Negative control cells were unlabeled with BrdU and treated as above. Labeled cells were pelleted by centrifugation at 200× g for 10 min, resuspended in 1 ml ice-cold PBS containing 2% FBS, and analyzed with FACSCalibur flow cytometer and CellQuest software (BD Biosciences). For apoptosis assay, SV cells were plated at 5×10^5^ cells/35 mm dish, treated for 48 hr with adipogenic inducers 4 days after plating, and analyzed 20 hr later for apoptosis by flow cytometry using Annexin V-FITC apoptosis detection kit (MBL).

### Statistical tests

Statistical significance was tested using an unpaired Student's *t* test or one-way ANOVA followed by Tukey's *post hoc* test. A significance of *P*<0.05 was required for rejection of the null hypothesis.

## Supporting Information

Figure S1 **Recombinant lentivirus vector for shRNA-based necdin knockdown.** (*A*) Schematic illustration of lentivirus vector expressing necdin-specific shRNA. EF-1α, elongation factor-1α; EmGFP, Emerald Green Fluorescent Protein; shRNA; short-hairpin RNA expression region; WPRE, Woodchuck hepatitis virus posttranscriptional regulatory element; CMV-R-U5, cytomegalovirus promoter +R/U5 region; DU3-LTR, deleted U3 region of the 5′ long terminal repeat of human immunodeficiency virus type 1. (*B, C*) Western blot analysis of endogenous necdin. Necdin in cultured adipose SV cells *in vitro* (*B*) and interscapular WAT *in vivo* (*C*) infected with lentiviruses expressing control RNA (cRNA) and necdin shRNA (shRNA) was analyzed by Western blotting. Molecular sizes are in kilodaltons (kDa).
(TIF)

Figure S2 **Body weight gain, food intake, and body core temperature of** ***Ndn*** **^+/+^ and** ***Ndn*** **^+m/−p^ mice.** (*A*) Body weight curves. Mice fed the high-fat diet were weighed weekly from 7 to 16 weeks of age (mean ± SEM, *n* = 3). (*B*) Food intake. *Ndn* ^+/+^ and *Ndn* ^+m/−p^ mice were fed the standard (SD) and high-fat (HD) diets from 5 to 14 weeks of age. Total food amounts were measured weekly. Data are presented as mean ± SEM (SD, *n* = 6 for *Ndn* ^+/+^, *n* = 5 for *Ndn* ^+m/−p^; HD, *n* = 5 for *Ndn* ^+/+^, *n* = 7 for *Ndn* ^+m/−p^). (*C*) Core body temperature. Rectal temperatures of *Ndn* ^+/+^ and *Ndn* ^+m/−p^ mice at 5 (*n* = 10–11) and 12 weeks (wk)(*n* = 4) of age were measured. No statistically significant differences were noted between *Ndn* ^+/+^ and *Ndn* ^+m/−p^ mice (*B*, *C*).
(TIF)

Figure S3 **Expression profiles of energy metabolism-related genes in the muscle and liver.** (*A*) Necdin mRNA levels in the brain, skeletal muscle, and liver. (*B*, *C*) Expression of energy metabolism-related genes in the skeletal muscle and liver. Total RNA was extracted from the brain, muscle, and liver of 5-week-old mice. qRT-PCR was performed using primers listed in Table S1. The values in the muscle (*B*) and liver (*C*) were normalized with those of β-actin and β-2 microglobulin, respectively. All data are presented as mean ± SEM (*n* = 4). No statistically significant differences were noted between *Ndn* ^+/+^ and *Ndn* ^+m/−p^ mice (*B*, *C*). PPAR, peroxisome proliferators-activated receptor; aP2, adipocyte fatty acid binding protein; Cox2, cyclooxygenase 2; GLUT4, glucose transporter type 4; SREBP, sterol regulatory element-binding protein; Ppargc1a, peroxisome proliferators-activated receptor coactivator 1α; UCP, uncoupling protein; Cyt-c, cytochrome c.
(TIF)

Figure S4 **Adiposity is enhanced in** ***Ndn*** **^+m/−p^ mice fed the high-fat diet.** (*A, B*) Dorsal (*A*) and ventral (*B*) views of *Ndn* ^+/+^ and *Ndn* ^+m/−p^ littermates. Mice were fed the high-fat diet from 5 to 14 weeks of age. IS, interscapular WAT pad; IG, inguinal WAT pad; EP, epididymal WAT pad. Scale bars, 1 cm (*A*, *B*).
(TIF)

Figure S5 **Expression of marker genes in adipose SV fraction.** (*A–D*) qRT-PCR. Total RNA was extracted from the SVF, adipocyte fraction (AF), pooled WAT (WAT) and interscapular BAT (BAT) in 5-week-old mice. Relative expression levels (SVF level = 1) of mRNAs encoding CD34 (*A*), PPARγ2 (*B*), aP2 (*C*), and UCP1 (*D*) were analyzed by qRT-PCR using specific primers listed in Table S1.
(TIF)

Figure S6 **Changes in cell number during adipogenic induction of the SV cells.** SV cells were prepared from *Ndn* ^+/+^ and *Ndn* ^+m/−p^ littermates and plated at 5×10^5^ cells/35 mm dish. Cells were grown for 96 hr to reach confluence and treated with adipogenic inducers as indicated by the arrow. Cells were trypsinized and counted at the time points indicated (mean ± SEM, *n* = 3). *P<0.05, **P<0.01 (*Ndn* ^+/+^ vs. *Ndn* ^+m/−p^).
(TIF)

Table S1 **Primer sequences used for qRT-PCR.**
(DOC)
