# Supplementary figures and images for: Necdin Controls Proliferation of White Adipocyte Progenitor Cells
Source: PLoS One. 2012 Jan 23;7(1):e30948. doi: 10.1371/journal.pone.0030948 (PMC3264651; doi:10.1371/journal.pone.0030948)

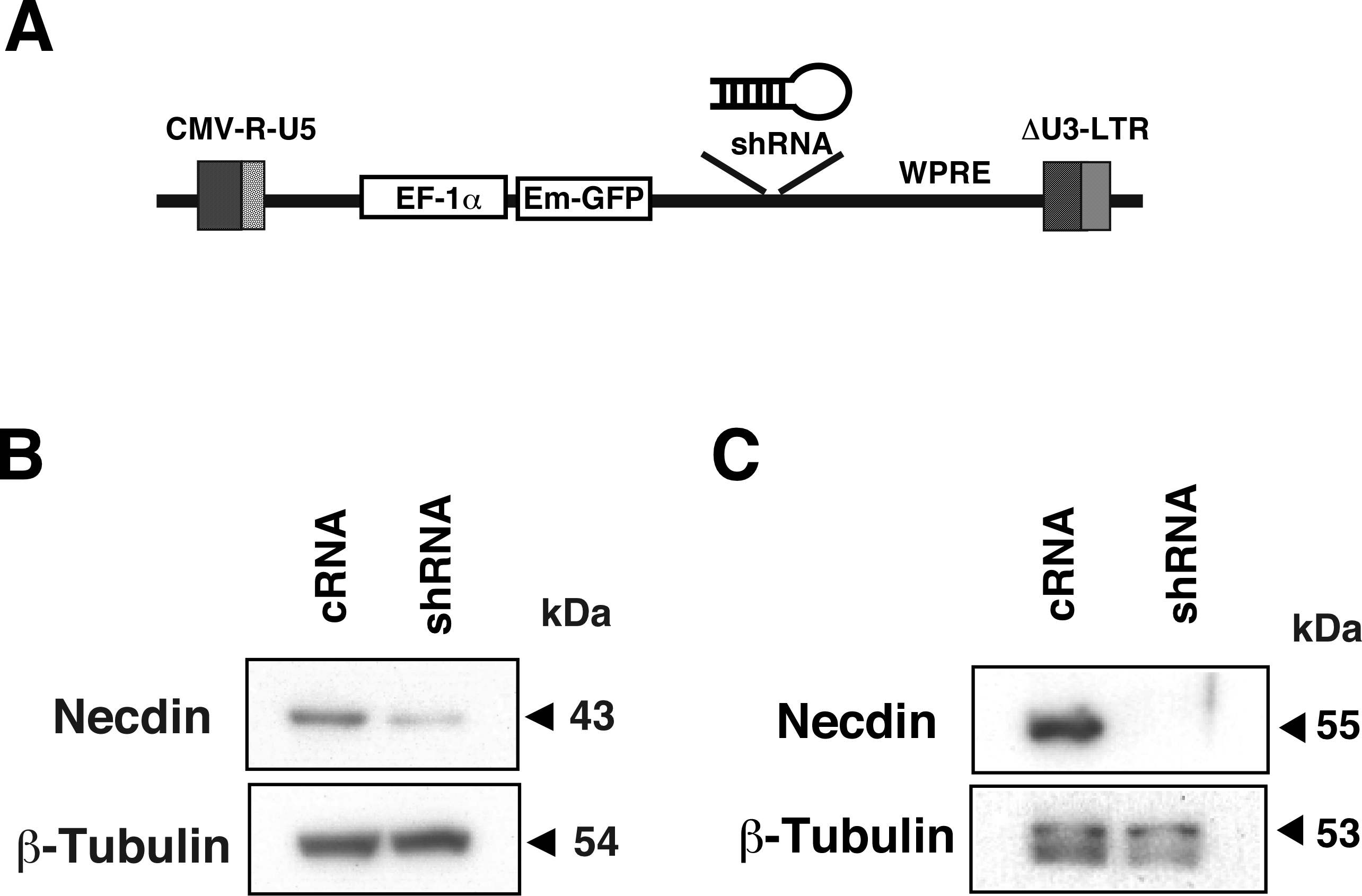

Supplement: Figure S1 — Recombinant lentivirus vector for shRNA-based necdin knockdown. (A) Schematic illustration of lentivirus vector expressing necdin-specific shRNA. EF-1α, elongation factor-1α; EmGFP, Emerald Green Fluorescent Protein; shRNA; short-hairpin RNA expression region; WPRE, Woodchuck hepatitis virus posttranscriptional regulatory element; CMV-R-U5, cytomegalovirus promoter +R/U5 region; DU3-LTR, deleted U3 region of the 5′ long terminal repeat of human immunodeficiency virus type 1. (B, C) Western blot analysis of endogenous necdin. Necdin in cultured adipose SV cells in vitro (B) and interscapular WAT in vivo (C) infected with lentiviruses expressing control RNA (cRNA) and necdin shRNA (shRNA) was analyzed by Western blotting. Molecular sizes are in kilodaltons (kDa). (TIF) [file pone.0030948.s001.tif]

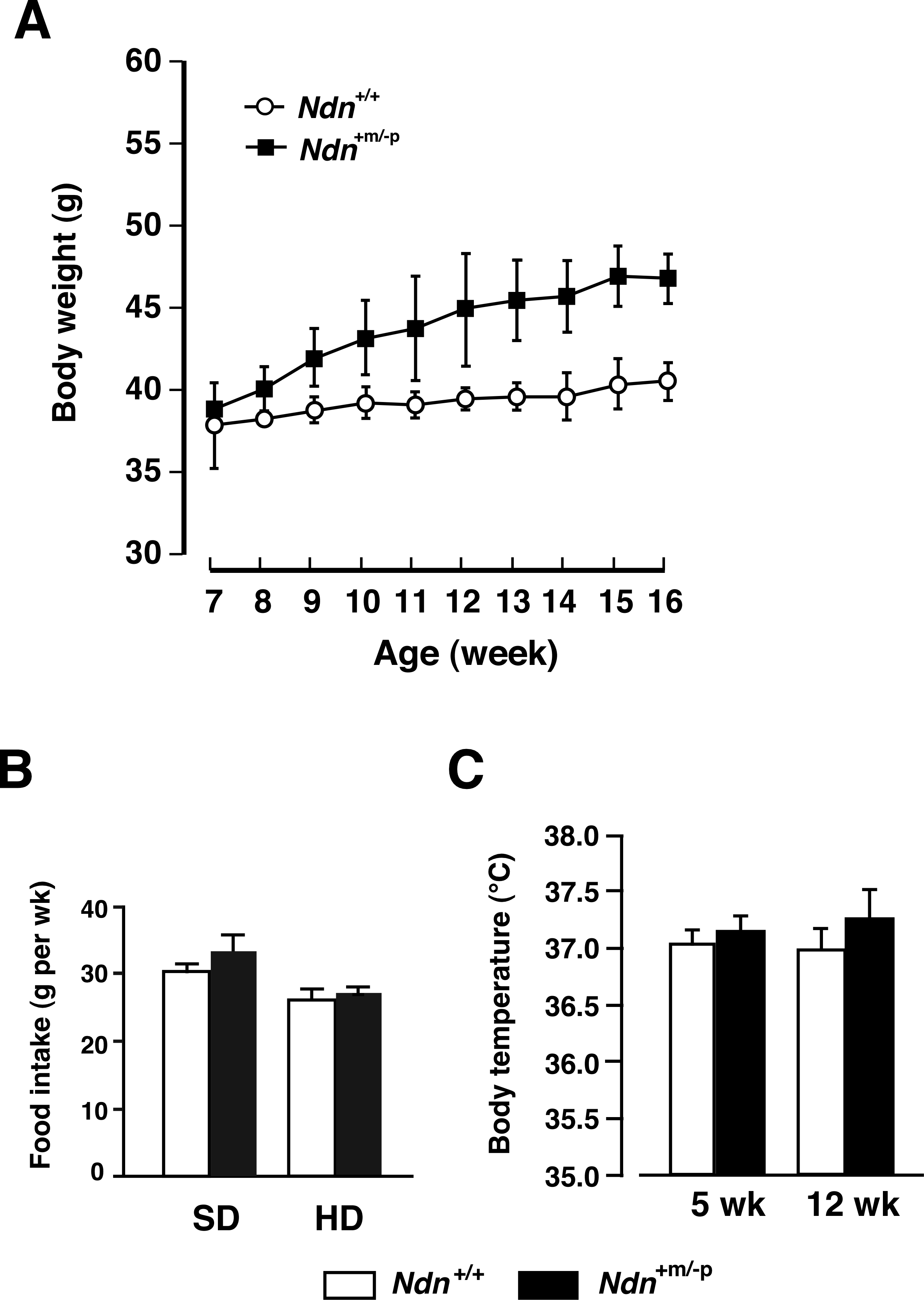

Supplement: Figure S2 — Body weight gain, food intake, and body core temperature of Ndn +/+ and Ndn +m/−p mice. (A) Body weight curves. Mice fed the high-fat diet were weighed weekly from 7 to 16 weeks of age (mean ± SEM, n = 3). (B) Food intake. Ndn +/+ and Ndn +m/−p mice were fed the standard (SD) and high-fat (HD) diets from 5 to 14 weeks of age. Total food amounts were measured weekly. Data are presented as mean ± SEM (SD, n = 6 for Ndn +/+, n = 5 for Ndn +m/−p; HD, n = 5 for Ndn +/+, n = 7 for Ndn +m/−p). (C) Core body temperature. Rectal temperatures of Ndn +/+ and Ndn +m/−p mice at 5 (n = 10–11) and 12 weeks (wk)(n = 4) of age were measured. No statistically significant differences were noted between Ndn +/+ and Ndn +m/−p mice (B, C). (TIF) [file pone.0030948.s002.tif]

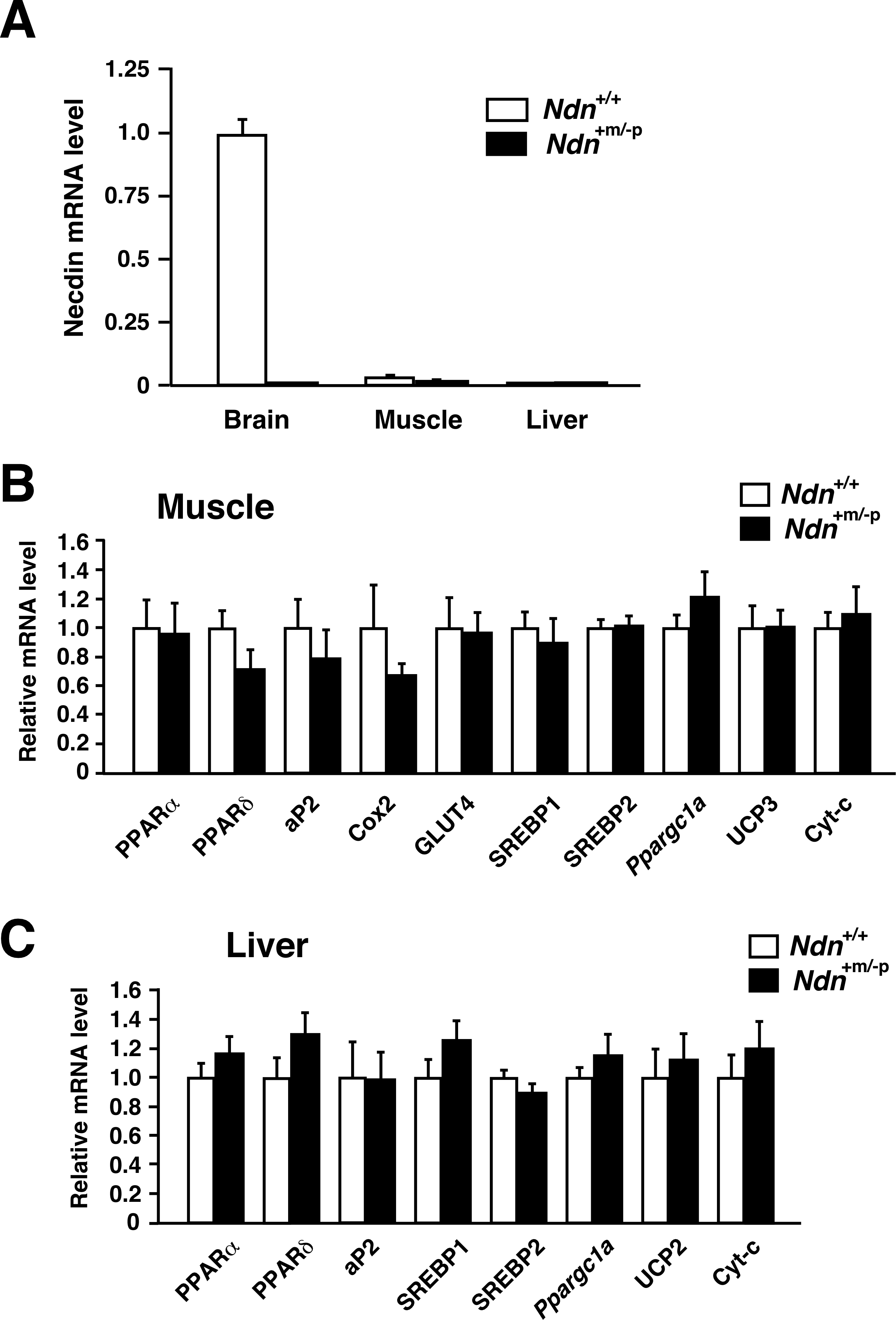

Supplement: Figure S3 — Expression profiles of energy metabolism-related genes in the muscle and liver. (A) Necdin mRNA levels in the brain, skeletal muscle, and liver. (B, C) Expression of energy metabolism-related genes in the skeletal muscle and liver. Total RNA was extracted from the brain, muscle, and liver of 5-week-old mice. qRT-PCR was performed using primers listed in Table S1. The values in the muscle (B) and liver (C) were normalized with those of β-actin and β-2 microglobulin, respectively. All data are presented as mean ± SEM (n = 4). No statistically significant differences were noted between Ndn +/+ and Ndn +m/−p mice (B, C). PPAR, peroxisome proliferators-activated receptor; aP2, adipocyte fatty acid binding protein; Cox2, cyclooxygenase 2; GLUT4, glucose transporter type 4; SREBP, sterol regulatory element-binding protein; Ppargc1a, peroxisome proliferators-activated receptor coactivator 1α; UCP, uncoupling protein; Cyt-c, cytochrome c. (TIF) [file pone.0030948.s003.tif]

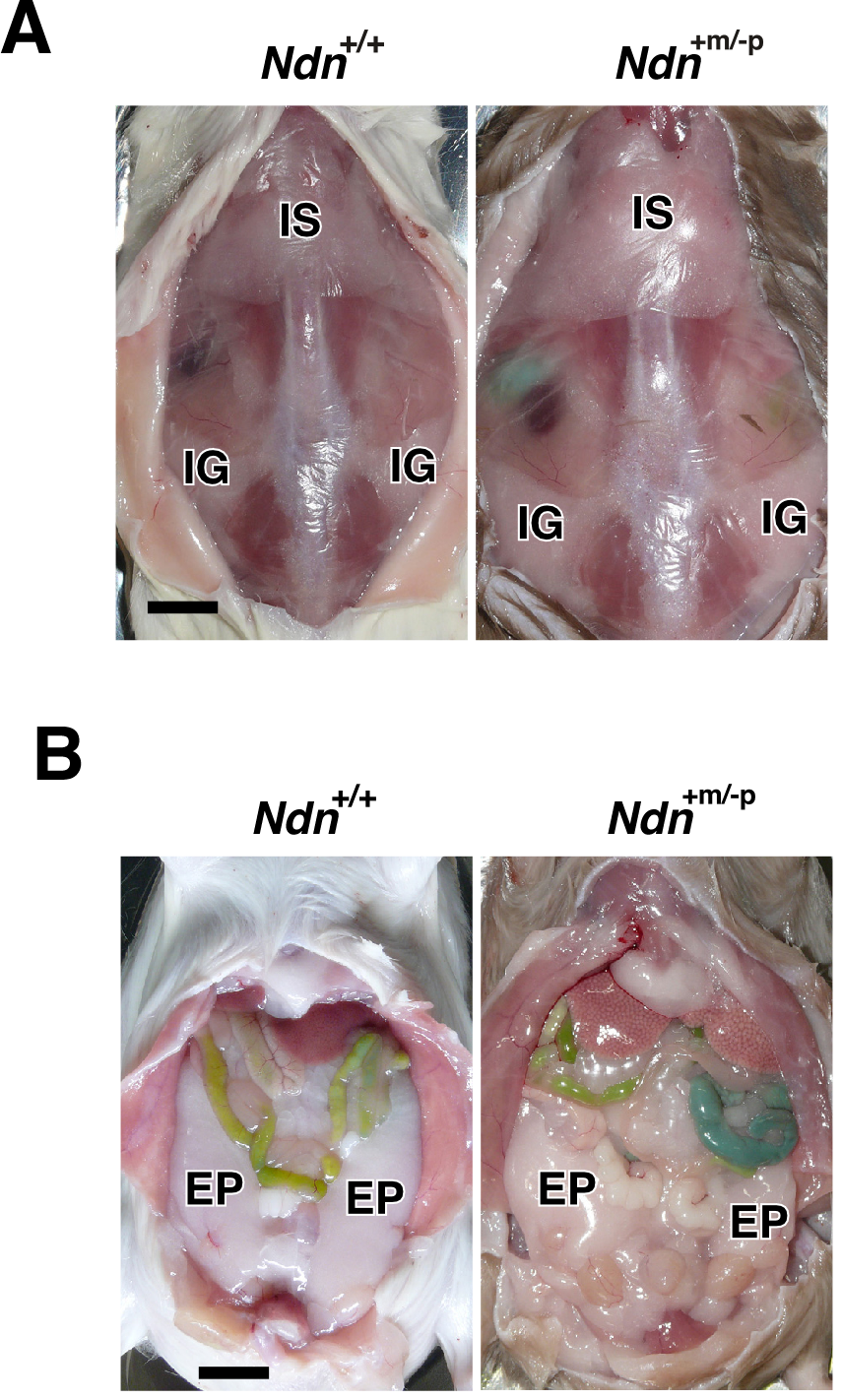

Supplement: Figure S4 — Adiposity is enhanced in Ndn +m/−p mice fed the high-fat diet. (A, B) Dorsal (A) and ventral (B) views of Ndn +/+ and Ndn +m/−p littermates. Mice were fed the high-fat diet from 5 to 14 weeks of age. IS, interscapular WAT pad; IG, inguinal WAT pad; EP, epididymal WAT pad. Scale bars, 1 cm (A, B). (TIF) [file pone.0030948.s004.tif]

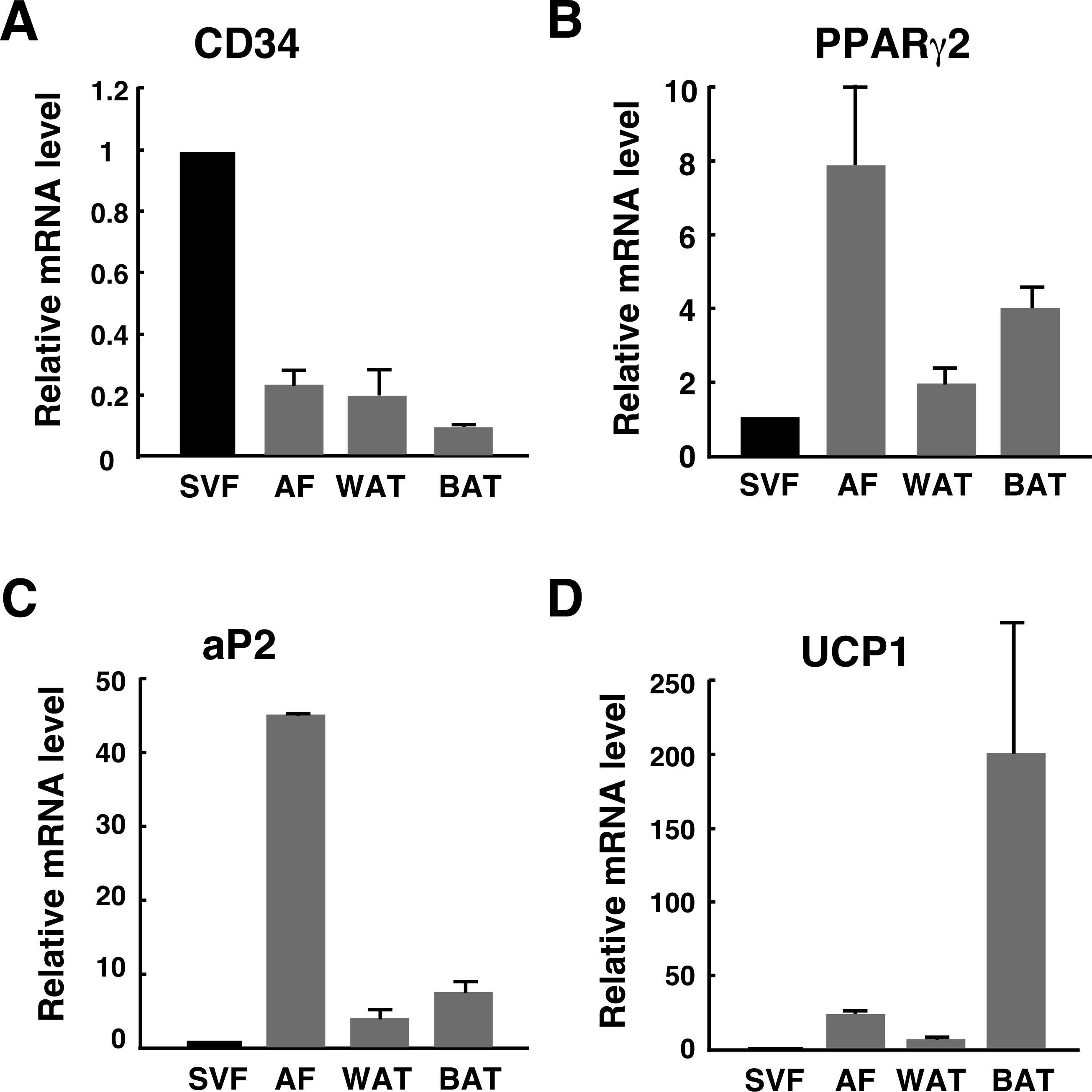

Supplement: Figure S5 — Expression of marker genes in adipose SV fraction. (A–D) qRT-PCR. Total RNA was extracted from the SVF, adipocyte fraction (AF), pooled WAT (WAT) and interscapular BAT (BAT) in 5-week-old mice. Relative expression levels (SVF level = 1) of mRNAs encoding CD34 (A), PPARγ2 (B), aP2 (C), and UCP1 (D) were analyzed by qRT-PCR using specific primers listed in Table S1. (TIF) [file pone.0030948.s005.tif]

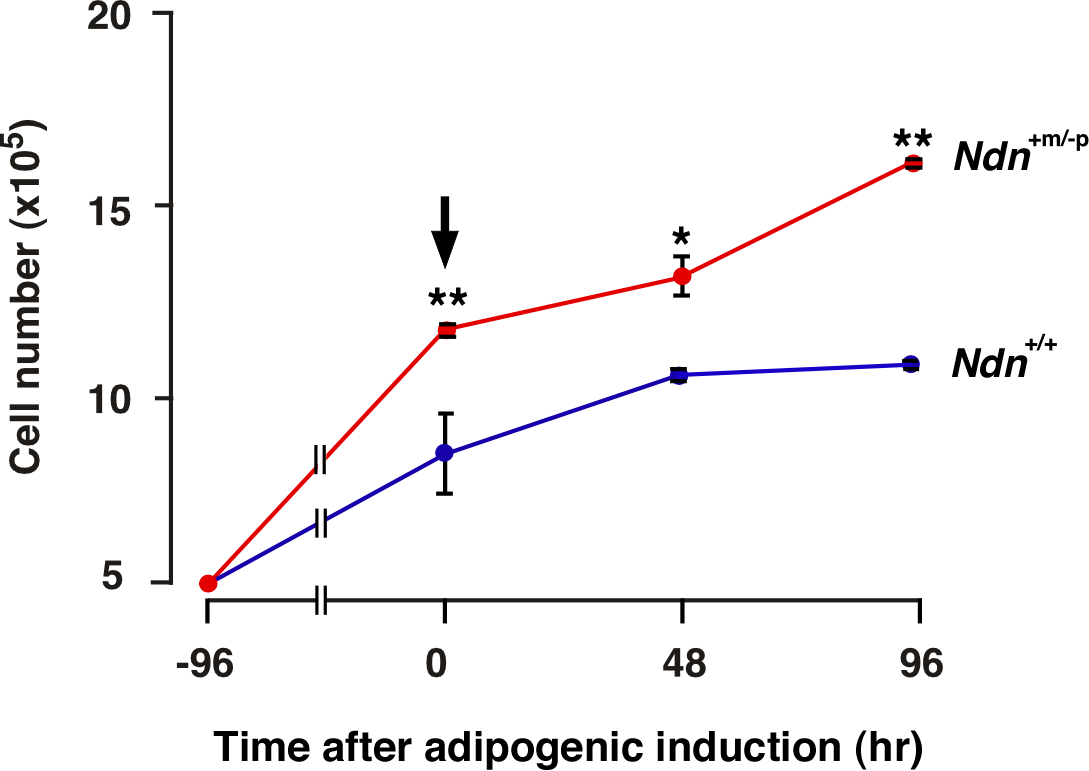

Supplement: Figure S6 — Changes in cell number during adipogenic induction of the SV cells. SV cells were prepared from Ndn +/+ and Ndn +m/−p littermates and plated at 5×10^5^ cells/35 mm dish. Cells were grown for 96 hr to reach confluence and treated with adipogenic inducers as indicated by the arrow. Cells were trypsinized and counted at the time points indicated (mean ± SEM, n = 3). *P<0.05, **P<0.01 (Ndn +/+ vs. Ndn +m/−p). (TIF) [file pone.0030948.s006.tif]
