# Supplementary material for: Necdin Controls Proliferation of White Adipocyte Progenitor Cells
Source: PLoS One. 2012 Jan 23;7(1):e30948. doi: 10.1371/journal.pone.0030948 (PMC3264651; doi:10.1371/journal.pone.0030948)
Supplement: Table S1 — Primer sequences used for qRT-PCR. (DOC) [file pone.0030948.s007.doc]

Table S1. Primer sequences used for qRT-PCR.

| Gene | Forward 5’ -> 3’ | Reverse 5’ -> 3’ |
| --- | --- | --- |
| *necdin* | AGGACCTGAGCGACCCTAAC | TGCTGCAGGATTTTAGGGTCAAC |
| *CD34* | ACCAGAGCTATTCCCGAAAG | AGCCTCTTTTCTTCCCAACA |
| *PPARγ2* | TTCGCTGATGCACTGCCTATG | CGCACTTTGGTATTCTTGGAGCTT |
| *aP2* | TGGAATTCGATGAAATCACC | TGCCCTTTCATAAACTCTTG |
| *UCP1* | CTTGTCAACACTTTGGAAAGG | CCTTGGTGTACATGGACATC |
| *PPARγ1/2* | CCATGAGATCATCTACACGA | CATTGAACTTCACAGCAAAC |
| *PPAR*α | CTGAAGCTGGTGTACGACAA | AGGTCGTGTTCACAGGTAAG |
| *PPARβ/*δ | TGAAGGCCTTCTCTAAGCAC | AACACGTGCACACTGATCTC |
| *Cox2* | CGTGGTCACTTTACTACGAG | GGGGAGGTACATAGTAGTCC |
| *GLUT4* | TGTTTTGAAGAACGGATAGG | CGGATTTCTTGAGTTCAAGG |
| *SREBP1* | CGTAGAGAAGCTTGGCGATCA | TCCAGATCTGCCACTAGAGGT |
| *SREBP2* | GACTGATTGTTCTGAGCTGTTACC | CCCCACATTGCTTCCAACTG |
| *Ppargc1a* | TATACTTTACGCAGGTCGAA | ACAGAGAGTGTAAAGTAGGAG |
| *UCP3* | ATGTGGTAAAGACCCGATAC | CACGGAGGACTAAAACTCTC |
| *Cyt-C* | AACCCATGAAGTACATGTGG | TGTAACGGAAGACAGATGGT |
| *UCP2* | AAAGATACTCTCCTGAAAGCC | CATAGGTGACAAACATCACTAC |
| *C/EBPα* | AGTTATGACAAGCTTCCCAA | AAAAGAATGAGAGCCTCACT |
| *adiponectin* | GAAAGGAGATGCAGGTCTTC | AAAGCGAATGGGTACATTGG |
| *GAPDH* | TCAACGACCCCTTCATTGAC | ATGCAGGGATGATGTTCTGG |
| *β-actin* | TGGAATCCTGTGGCATCCATGAAAC | TAAAACGCAGCTCAGTAACAGTCCG |
| *β-2-microglobulin* | GACCGTCTACTGGGATCGAG | GTAATGAGAAGTACAGAGGGTTTGG |
